# Case Report: ^18^F-FDOPA PET in the clinical management of a dog with an intraventricular tumor suspected to be choroid plexus papilloma

**DOI:** 10.3389/fvets.2025.1477063

**Published:** 2025-03-26

**Authors:** Juwon Wang, Yeon Chae, Dohee Lee, Taesik Yun, Hakhyun Kim, Byeong-Teck Kang

**Affiliations:** Laboratory of Veterinary Internal Medicine, College of Veterinary Medicine, Chungbuk National University, Cheongju, Republic of Korea

**Keywords:** canine, dog, ^18^F-FDOPA, choroid plexus papilloma, positron emission tomography

## Abstract

An 8-year-old neutered male Miniature Poodle, weighing 6.7 kg, was presented with lethargy, anorexia, and single seizure episode. Neurological examination revealed bilaterally absent menace reflexes and an obtunded mental status. Magnetic resonance imaging showed a papilliform shaped mass measuring 1.2 × 1.4 × 1.3 cm in size, with a volume of 1.17 cm^3^ in the third ventricle. 3,4-dihydroxy-6-[^18^F] fluoro-l-phenylalanine (^18^F-FDOPA) positron emission tomography/computed tomography (PET/CT) was performed 53 days after presentation, revealing a hypermetabolic region in the intraventricular mass with mean and maximal standardized uptake values (SUV_mean_ and SUV_max_) of 1.2 and 1.42, respectively, and a tumor to normal tissue (T/N) ratio of 1.33. The mass lesion measured 1.3 × 1.4 × 1.2 cm in size, with a volume of 1.09 cm^3^ on contrast-enhanced CT images. The metabolic tumor volume (MTV) was 1.184 ${\text{cm}}_{.}^{3}$ No evidence of brain parenchymal metastases was observed. Therefore, the dog was tentatively diagnosed with a brain tumor, which was suspected to be a choroid plexus papilloma (CPP) and chemotherapy with prednisolone and cyclophosphamide was initiated. As worsening clinical signs were observed, a second ^18^F-FDOPA PET/CT scan was performed on day 183. The SUV_mean_, SUV_max_, and T/N ratio of the lesion were 1.49, 1.85, and 1.62, respectively. The mass lesion measured 1.0 × 1.0 × 1.3 cm in size, with a volume of 0.68 cm^3^ on contrast-enhanced CT images, whereas the MTV was increased to 2.217 cm^3^. The dog died 186 days after the presentation. To the best of our knowledge, this is the first report describing the ^18^F-FDOPA PET/CT findings in a dog with an intraventricular brain tumor suspected of having CPP. In the present case, although the lesion size decreased on CT contrast imaging, an increase in the MTV was observed on follow-up ^18^F-FDOPA PET/CT after chemotherapy. Thus, an increase in MTV post-chemotherapy combined with the worsening clinical signs and limited survival period in dogs correlates with poor prognosis, as previously reported in a human study. This case offers significant diagnostic insights into canine intraventricular tumors within the field of veterinary medicine.

## Introduction

Intraventricular brain tumors in dogs mainly arise from the ependymal cells and the choroid plexus epithelium. Therefore, differential diagnoses for intraventricular tumors in dogs include choroid plexus tumors (CPT), ependymomas, oligodendrogliomas, and astrocytomas (1).

CPTs are mostly found in the intraventricular region, originating from the epithelium of the choroid plexus, wherein the primary mass is mostly located in the lateral or third ventricle (2). They constitute 10% of primary intracranial central nervous system tumors in dogs. Although the definitive diagnosis of CPT relies on histopathological examination, magnetic resonance imaging (MRI) and cerebrospinal fluid (CSF) analysis are used for presumptive clinical diagnosis of brain tumors in dogs. CPT is suspected based on MRI results of an intraventricular mass at or adjacent to anatomical sites of the choroid plexus. Commonly observed features include intraventricular masses characterized by prominent contrast enhancement and ventriculomegaly (3–8). Several treatment modalities are available for CPTs including surgery, radiation therapy, chemotherapy, and palliative treatment with anticonvulsants and steroids (9–14). Conversely, in veterinary medicine, chemotherapy protocols for CPTs remain limited, and a standardized treatment regimen has not been established; however, cyclophosphamide, one of the most effective agents for treating choroid plexus-derived tumors in human medicine is known to cross the blood-brain barrier (BBB) in humans, and may be a treatment option (15).

Positron emission tomography (PET) offers significant advantages over conventional imaging methods, such as computed tomography (CT) or MRI, which primarily focus on anatomical features, by providing functional information about tumors. One of the amino acid analogs used in PET is 3,4-dihydroxy-6-[^18^F] Fluoro-L-phenylalanine (^18^F-FDOPA) (16). ^18^F-FDOPA absorption is facilitated by up-regulated amino acid transporters, which is attributed to the nature of brain tumor (16, 17). Additionally, the efficacy of ^18^F-FDOPA PET (96%) surpasses that of ^18^F- Fluoro-2-Deoxyglucose PET (^18^F-FDG) PET (61%) in visualizing human brain tumors (18).

Previously, human studies have demonstrated the superiority of ^18^F-FDOPA over MRI in visualizing tumors more accurately and delineating brain tumor margins (19). Moreover, metabolic tumor volume (MTV) obtained from ^18^F-FDOPA images provides valuable insights into predicting tumor recurrence or progression, assessing treatment response, and predicting the prognosis of human brain tumors (17, 20, 21). However, only a single instance has been reported in dogs, wherein a tumor lesion was detected by ^18^F-FDG PET but was successfully identified through visual analysis using ^18^F-FDOPA PET, underscoring the superiority of ^18^F-FDOPA PET over ^18^F-FDG PET (22).

Herein, we present a report describing the application of ^18^F-FDOPA in visualizing an intraventricular brain tumor suspected to be a CPT, particularly choroid plexus papilloma (CPP), in a dog.

## Case description

An 8-year-old neutered male Miniature Poodle presented with a lethargy, anorexia, and tonic seizures. On physical examination, the dog weighed 6.7 kg, had a pulse rate of 114 beats per minute, a respiratory rate of 30 breaths per minute, and a rectal temperature of 38.3 °C. Neurological examination revealed bilaterally absent menace reflexes and an obtunded mental status. No other abnormalities were observed, and ocular findings were unremarkable. Complete blood count revealed thrombocytopenia (81 × 10^3^/μL; reference interval RI: 148–484 × 10^3^/μL), whereas serum biochemical analysis did not show any remarkable abnormalities, except for mildly decreased aspartate transaminase activity (18 mg/dL; RI: 23–66 mg/dL), hypotriglyceridemia (19 mg/dL; RI: 21–116 mg/dL), and hyperlactatemia (2.99 mmol/L; RI: 0.5–2.5 mmol/L). Blood electrolyte analysis revealed mild hypocalcemia (8.5 mg/dL; RI: 9–11.3 mg/dL) and mild hypomagnesemia (1.6 mg/dL; RI: 1.8–2.4 mg/dL), which were clinically insignificant.

**Figure 1:**
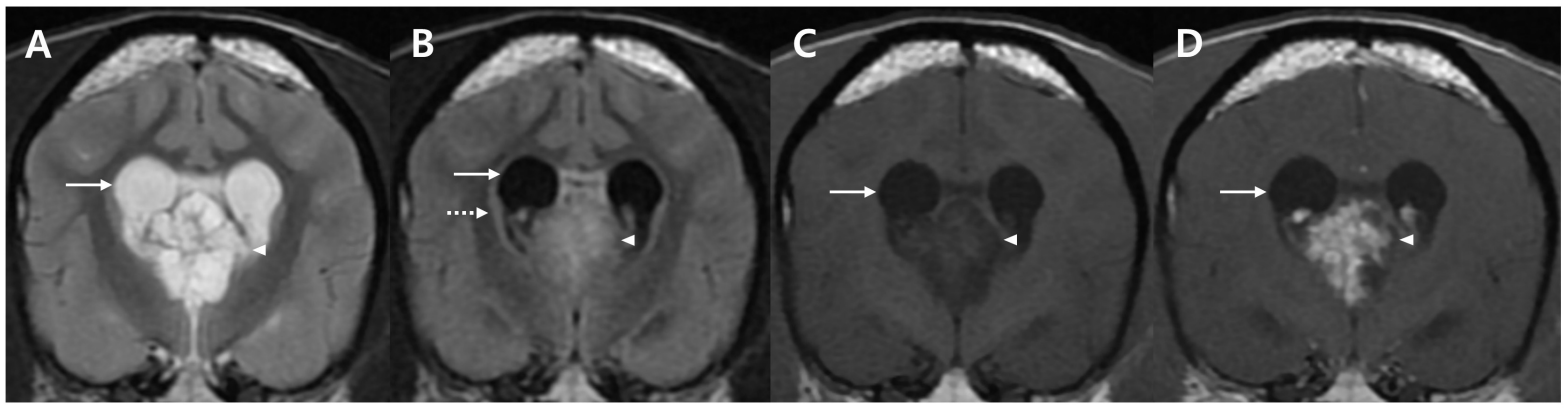
MRI characteristics of a dog with an intraventricular tumor suspected to be choroid plexus papilloma presented in the transverse plane. A well-defined solitary papilliform shaped mass is observed in the third ventricle with enlarged lateral ventricle (arrows). The tumor lesion (arrow heads) shows hyperintensity on T2-weighted images (WI) **(A)**, hyperintensity on FLAIR images **(B)** with periventricular edema (dotted arrow), and hypointensity on T1-WI **(C)**. Post-contrast T1-WI **(D)** image shows uniformly remarkable enhancement (arrow). No metastatic findings or brain parenchymal involvement were identified. MRI, magnetic resonance imaging; T1-WI, T1-weighted image; T2-WI, T2-weighted image; FLAIR, fluid-attenuated inversion recovery.

Based on clinical signs, neurological, ocular, and laboratory examination results, the lesion was neuroanatomically localized to the forebrain. A brain MRI was performed using 1.5-Tesla unit (Signa Creator; GE Healthcare, Milwaukee, WI, USA). The dog was anesthetized via intravenous administration of 6 mg/kg propofol (Provive, Myungmoon Pharm. Co., Ltd, Seoul, South Korea) and 0.2 mg/kg midazolam (Midazolam, Bukwang Pharm. Co., Ltd., Seoul, South Korea) and maintained by inhalation of 2.0–2.5% isoflurane (Terrell, Piramal Critical Care, Bethlehem, PA, USA) in 100% oxygen in a circle rebreathing circuit. T1-weighted images (WI) (pre- and post-contrast), T2-WI, and fluid-attenuated inversion recovery (FLAIR) images were obtained using transverse, sagittal, and dorsal planes. A papilliform shaped mass lesion measuring 1.2 × 1.4 × 1.3 cm in size, with a volume of 1.17 cm^3^ was detected in the third ventricle. Additionally, periventricular and peritumoral edema were observed around the lesion (Figure 1). The papilliform-shaped mass was identified as hyperintense on T2-WI (Figure 1A) and FLAIR images (Figure 1B) and hypointense on T1-WI (Figure 1C). A remarkable enhancement in the papilliform shaped mass on T1-WI was noted after administration of 0.1 mmol/kg gadolinium-diethylenetriamine pentaacetic acid [IV; OmniscanTM, GE Healthcare (Shanghai), Co., Ltd, China], (Figure 1D). However, diagnosis and treatment options involving a surgical approach for the lesion could not be performed due to lack of consent from the owner. Nonetheless, a diagnosis of CPP was strongly suspected based on the patient's history, clinical assessments, and MRI features, even though CSF collection and analysis could not be performed owing to considerations regarding post-seizure intracranial pressure elevation.

**Figure 2:**
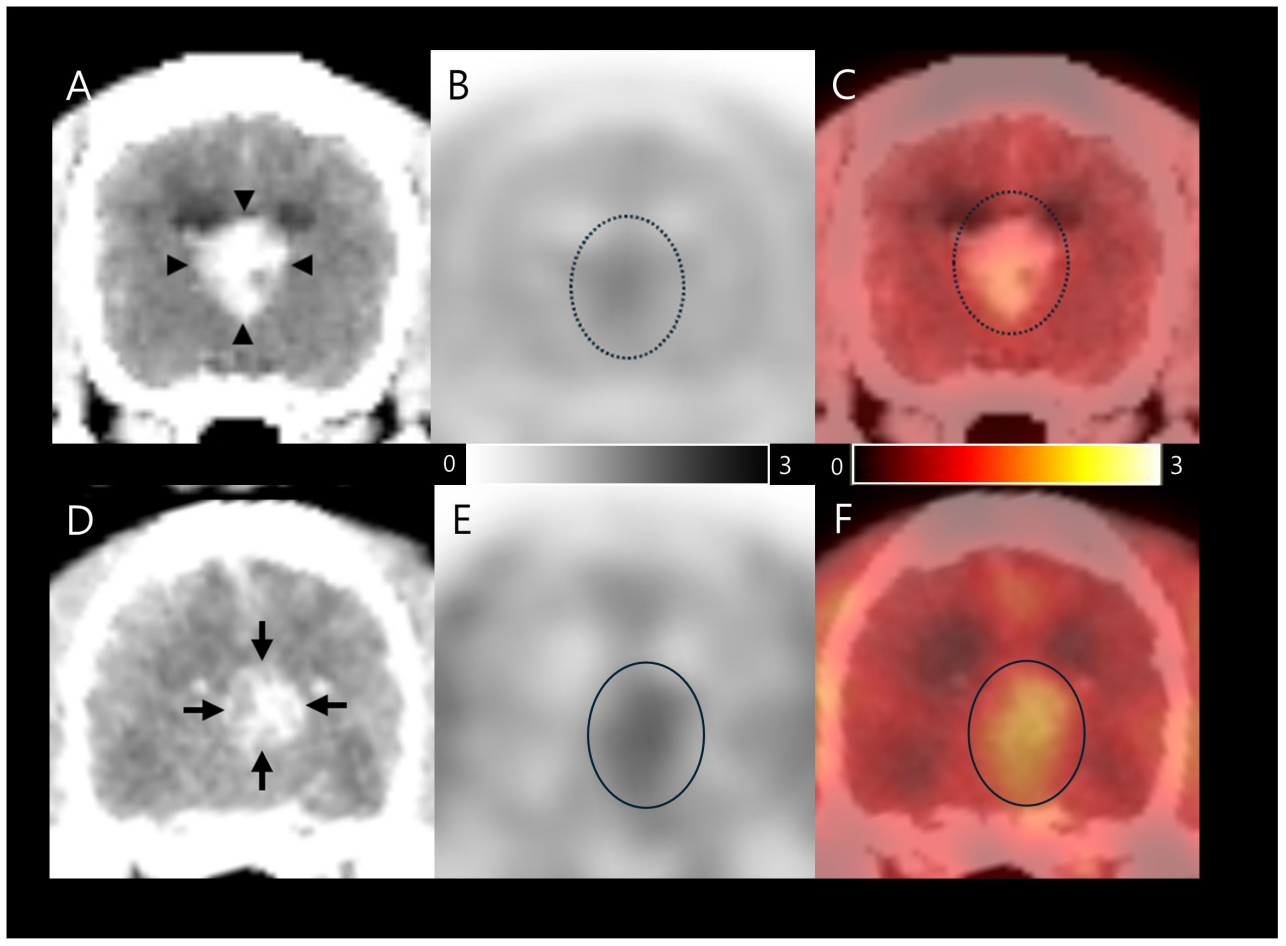
^18^F-FDOPA PET/CT findings in a dog with an intraventricular tumor suspected to be choroid plexus papilloma. The first image was taken on day 53, after initial symptomatic therapy **(A–C)**; Contrast-enhanced CT **(A)** image shows size of 1.3 × 1.4 × 1.2 cm papilliform shaped mass (arrow heads). ^18^F-FDOPA PET **(B)** and PET/CT fusion **(C)** images showed elevated ^18^F-FDOPA uptake (dotted circles) with SUV_mean_ 1.2, SUV_max_ 1.42, T/N ratio 1.33, and MTV 1.184 cm^3^. The second image was captured on day 117 after initiation of chemotherapy; Contrast-enhanced CT **(D)** image shows size of 1.0 × 1.0 × 1.3 cm papilliform shaped mass (arrows). ^18^F-FDOPA PET **(E)** and PET/CT fusion **(F)** images demonstrated remarkable elevated ^18^F-FDOPA uptake in the papilliform shaped tumor lesion (circles) with SUV_mean_ 1.49, SUV_max_ 1.62, T/N ratio 1.62, and MTV 2.217 cm^3^. The black and white scale bar represents high ^18^F-FDOPA uptake in black and low uptake in white (B and E), while the color scale bar represents high ^18^F-FDOPA uptake in yellow and low uptake in red **(C, F)**. ^18^F-FDOPA, 3,4-dihydroxy-6-[^18^F]-fluoro-l-phenylalanine; PET, positron emission tomography; CT, computed tomography; SUV, standard uptake value; T/N, tumor to normal tissue; MTV, metabolic tumor volume.

Therefore, symptomatic therapy was prescribed with prednisolone 0.5 mg/kg PO q12h (Solondo^®^, Yuhan, Seoul, South Korea) and zonisamide 10 mg/kg PO q12h (Excegran^®^, Dong-A, Seoul, South Korea). After 53 days, an ^18^F-FDOPA PET scan of whole body, including the head, was performed to determine malignancy of the tumor and whether metastasis had occurred. ^18^F-FDOPA (0.094 mCi/kg) was intravenously administered into the right saphenous vein, followed by 5 mL of 0.9% normal saline to flush residual ^18^F-FDOPA. CT images (pre- and post-contrast) were acquired before PET scans. Attenuation correction for PET image reconstruction was performed using pre-contrast CT images to prevent potential artifacts from iodine-based contrast agents. The PET scans (Discovery-STE, General Electric Medical Systems, Waukesha, WI, USA) were obtained 10 min after ^18^F-FDOPA injection (Figures 2A-C) and were analyzed using OsiriX MD v10.0 (Pixmeo Sarl, Geneva, Switzerland).

Based on visual evaluation of the contrast enhanced CT images, the papilliform shaped mass lesion measured 1.3 × 1.4 × 1.2 cm in size, with a volume of 1.09 cm^3^ (Figure 2A). An ^18^F-FDOPA avid tumor was identified in PET and PET/CT images, with no evidence of metastatic lesions (Figures 2B, C). The regions of interest (ROIs) were manually drawn on the PET/CT fusion images (Figure 2C). The metabolic activity of ROIs was converted to a standard uptake value (SUV) as follows: SUV = concentration of ^18^F-FDOPA in the ROIs (mCi/kg)/injected dose (mCi) per kilogram of body weight (kg). The mean and maximum SUVs (SUV_mean_ and SUV_max_) of the tumor were 1.2 and 1.42, respectively. The tumor to normal tissue (T/N) ratio was calculated by dividing the SUV_max_ of the tumor by the SUV_max_ of the normal brain parenchyma using the dorsal plane to evaluate metabolic activity objectively, and was 1.33. All voxels with SUV above the threshold were included to evaluate the MTV based on an SUV threshold determined by the SUV_mean_ of the normal tissue background (17). The MTV was calculated to be 1.184 cm^3^. The increase in intracranial pressure was expected to stabilize after medication. Eventually, with the owner's consent, CSF samples were collected via the foramen magnum immediately after the ^18^F-FDOPA PET scan for differential diagnosis. Cytological evaluation of CSF was conducted within 30 min of collection, with total nucleated cell counts, RBC counts, and differential nucleated cell counts assessed using a standard hemocytometer and methylene blue staining. Total protein concentration was measured using an automated biochemical analyzer (Catalyst One, IDEXX Laboratories, USA). CSF cytology analysis revealed no detectable nucleated cells or red blood cells, with a total protein concentration of 30 mg/dL. Polymerase chain reaction was negative for the following infectious agents: *Bartonella spp., Blastomyces dermatitidis, Coccidioides spp., Cryptococcus spp., Histoplasma capsulatum*, Canine distemper virus, West Nile virus, *Borrelia burgdorferi, Neospora spp., and Toxoplasma gondii*.

On initial evaluation, the dog was suspected to have CPP based on history, clinical assessments, and MRI features. CPP was more strongly presumed to be a tentative diagnosis following additional examinations, including CSF analysis and ^18^F-FDOPA PET. Chemotherapy with cyclophosphamide 12.9 mg/m^2^ PO q12 h, PO (Alkyroxan^®^, JW-pharma, Seoul, South Korea) and prednisolone 0.5 mg/kg PO q24 h was initiated based on its established efficacy in human cases, its accessibility in veterinary practice, and the owner's decision to proceed with this treatment option (15). Fifteen days after the commencement of chemotherapy, elevated liver enzyme levels were observed without worsening neurological signs, and prednisolone was tapered to 0.5 mg/kg q48 h. However, 100 days after the initiation of chemotherapy, dullness was observed, representing the first deterioration in neurological signs since the commencement of chemotherapy. Consequently, the prednisolone dose was increased to 0.5 mg/kg q24 h. On day 102 after the commencement of chemotherapy, cluster seizures were observed, and prednisolone dose was increased to 0.5 mg/kg q12 h. Additionally, potassium bromide was loaded at 100 mg/kg PO q12h for 2 days and tapered to 17.5 mg/kg PO q12 h as an epileptic drug to control cluster seizures.

A second ^18^F-FDOPA PET scan was performed to assess the treatment response after 117 days of chemotherapy (Figures 2D–F), employing the same procedure as previously described. A visual evaluation of the contrast enhanced CT image showed that the papilliform shaped mass lesion measuring 1.0 × 1.0 × 1.3 cm in size, with a volume of 0.68 cm^3^ was present in the third ventricle (Figure 2D). An ^18^F-FDOPA avid tumor was identified in the second PET/CT image, without evidence of metastatic lesions (Figures 2E, F). The tumor displayed an SUV_mean_ of 1.49 and SUV_max_ of 1.85. In addition, the T/N ratio was 1.62, and the MTV was 2.217 cm3. An increase in both ^18^F-FDOPA avidity and MTV was observed compared with the initial pre-chemotherapy scan results. Unfortunately, the dog died 120 days after the commencement of chemotherapy and 186 days after the initial presentation.

## Discussion

In the present case, the dog presented with an intraventricular brain tumor, which was suspected to be CPP, and survived for 186 days after diagnosis and 120 days after chemotherapy with prednisolone and cyclophosphamide. Unfortunately, necropsy could not be performed due to the owner's refusal, therefore, a definitive diagnosis could not be made. Nonetheless, a tentative diagnosis was made based on history, signalment, clinical assessment, MRI features, CSF analysis, and ^18^F-FDOPA PET scans. This is the first report describing ^18^F-FDOPA PET findings in a dog presenting with an intraventricular tumor suspected to be CPP. Our case suggests that the findings from ^18^F-FDOPA PET scans may provide superior clinical insights compared with those from conventional diagnostic methodologies.

Differentiation between CPP, choroid plexus carcinoma (CPC), meningioma, ependymoma, oligodendroglioma, and astrocytoma is essential (1). Both MRI and CSF examinations present distinct characteristics which help differentiate between CPC and CPP. CPPs typically present as a papilliform or globular mass on MRI, with variable T1-WI, contrast enhancement, and hyperintense T2-WI, often accompanied by periventricular edema. In contrast, CPCs exhibit MRI characteristics similar to those of CPP but may display multiform or linear shapes and occasionally metastasize. Additionally, the median CSF protein concentration for CPP is 34 mg/dL, ranging from 32 to 80 mg/dL, whereas that for CPC is 108 mg/dL, ranging from 27 to 380 mg/dL (2). Moreover, discrimination between CPPs and CPCs can be achieved by assessing CSF protein concentration, with a sensitivity of 67% and specificity of 100% when using a threshold of 80 mg/dL (2). Conversely, intraventricular meningiomas are rare, with only one documented case displaying a well-defined mass with T1 isointensity and T2 hyperintensity within the fourth ventricle (23). Whereas ependymomas typically manifest as smooth or lobulated masses on MRI, showing T1 isointensity, T2 hyperintensity, and variable contrast enhancement. Although parenchymal tumors have been described as breaking through the ependymal lining to invade the ventricular space, intraventricular occurrences of oligodendrogliomas are not well documented in veterinary literature (1). In the present case, MRI revealed a papilliform lesion in the third ventricle, demonstrating T1 hypointensity, T2 hyperintensity, and contrast enhancement without metastasis. Parenchymal lesions were not identified, and no signs of ventricular penetration were observed. The CSF protein concentration was 30 mg/dL, below the 80 mg/dL threshold for distinguishing CPP from CPC. Therefore, the patient was tentatively diagnosed with CPP based on these findings, however, a definitive diagnosis remained inconclusive owing to the lack of histopathological examination.

In the field of human oncology, PET/CT is extensively employed to assess tumor metabolism, metastases, and evaluate residual disease after radiotherapy and surgery (24–26). ^18^F-FDG, a glucose analog, is a commonly used PET tracer; however, the inherent high physiological glucose metabolism rate of normal brain tissue represents challenge for FDG PET scans in identifying intracranial malignancies (27). In contrast, ^18^F-FDOPA, an amino acid analog tracer, is advantageous for brain tumor imaging because of its low uptake in normal brain tissue and high uptake within tumor tissue. A previous human study reported that the sensitivity of ^18^F-FDOPA for the detection of brain tumors was higher than that of ^18^F-FDG (96% and 61%, respectively) (18, 28).

The detection of brain tumors using ^18^F-FDOPA has also been reported in veterinary medicine (22, 29). A previous case study involving canine glioma highlighted discrepancies between diagnostic results of ^18^F-FDG and ^18^F-FDOPA scans (22). Notably, although the tumor lesion was not detected on the ^18^F-FDG scan, it was confirmed on the ^18^F-FDOPA PET scan, which aligns with previous findings in human medicine (17, 29). Although ^18^F-FDOPA PET can detect tumor lesions with high sensitivity (18, 28), no metastatic tumor lesions were identified on ^18^F-FDOPA PET in this case. This observation serves as a further point of differentiation from CPC, where metastatic occurrences are prevalent in over half of cases (2).

The findings from the ^18^F-FDOPA PET scan in the current case suggest several implications. First, ^18^F-FDOPA PET scans could be used to detect not only brain parenchyma tumors, previously identified in veterinary medicine, but also intraventricular tumors. Second, ^18^F-FDOPA scans, which are highly sensitive to tumor detection, can determine the presence of metastasis to the brain parenchyma and serve as a distinguishing feature of the tumor. Third, the T/N ratio of the ^18^F-FDOPA PET scan, which serves as a diagnostic parameter for brain tumors, has demonstrated high sensitivity (96%) and specificity (86%) for identifying brain tumors in humans when its value exceeds 1.3 (18). The T/N ratio in the present case was 1.33 before chemotherapy, which is consistent with the cut-off value reported in human brain tumors; however, it increased to 1.62 at ^18^F-FDOPA PET/CT follow-up, which was conducted 117 days after chemotherapy with prednisolone and cyclophosphamide. Although it was challenging to diagnose and initiate chemotherapy for the tumor solely based on the results of conventional diagnostic methodologies, such as physical examination, neurological assessment, blood tests, MRI scans, and CSF analysis, with the utilization of T/N ratio results, a provisional diagnosis of brain tumor was made, allowing for the initiation of chemotherapy. Thus, differentiating between tumor and other intracranial conditions is crucial for developing treatment plans for veterinary patients presenting with brain abnormalities. Although biopsy remains an essential diagnostic tool for differential diagnosis in veterinary patients with brain lesions, its invasiveness and potential for complications limit its use. In such cases, ^18^F-FDOPA PET scans are a noninvasive diagnostic modality for identifying of brain tumors, including metastasis to brain parenchyma. Thus, our findings serve as important points for differentiating between different diagnoses, thereby effectively guiding the course of treatment. However, while ^18^F-FDOPA PET provides valuable metabolic insights, its limitations in distinguishing benign from malignant tumors and the lack of established evidence for detecting extracranial metastases suggest that it should not be solely relied upon for diagnosis. Consequently, a comprehensive evaluation incorporating other diagnostic modalities is essential (18).

Additionally, the MTV on ^18^F-FDOPA PET increased from 1.184 cm^3^ to 2.217 cm^3^ despite observed tumor volume upon MRI being 1.17 cm^3^ and on contrast-enhanced CT scans decreasing from 1.09 cm^3^ to 0.68 cm^3^ in the present case. A study in human medicine suggested that the response rate of MTV at follow-up examinations may serve as a prognostic indicator for brain tumor chemotherapy (17). This association is due to the increased amino acid uptake by the tumor, which may be due to the impairment of BBB integrity, suggesting tumor-induced BBB disruption (17). Moreover, this indicates that the normalization of BBB permeability leads to a decrease in the MTV in cases where the treatment response is favorable, whereas BBB permeability remains abnormal in cases where the treatment response is unfavorable (16). Although the median survival time for CPP is not well known due to the limited number of reports, a survival period of 388 days has been reported for dogs treated with lomustine and hydroxyurea (30). In addition, a survival of 15 months with symptomatic treatment alone has been reported (31). In this case, the dog died 186 days after diagnosis and 120 days after the start of chemotherapy. This coincides with a previously reported human study showing that non-responder MTV after treatment indicates a poor prognosis. Therefore, follow-up ^18^F-FDOPA PET scans could be helpful for monitoring treatment effects and evaluating prognosis after the treatment of intracranial tumors in veterinary medicine.

To the best of our knowledge, this is the first report to describe ^18^F-FDOPA PET findings in a clinical case of a dog with an intraventricular brain tumor suspected to have CPP. This case offers significant diagnostic insights into canine intraventricular tumors within the field of veterinary medicine. Nevertheless, further studies are required to establish diagnostic criteria using ^18^F-FDOPA PET scans, such as SUV_max_ or T/N ratio cut-off, for canine intraventricular tumors. In the present case, although the lesion size decreased on follow-up CT contrast imaging, an increase in the MTV was observed on the ^18^F-FDOPA PET/CT after chemotherapy. Consequently, integrating findings from other diagnostic modalities is important for a more comprehensive evaluation. Combining this with worsening clinical signs and a limited survival period suggests that increased MTV post-chemotherapy correlates with poor prognosis, as reported in human studies. Thus, MTV measurements from pre- and post-chemotherapy ^18^F-FDOPA scans could be valuable prognostic factors beyond lesion size assessments from contrast-enhanced CT images. However, this was a single case study; therefore, investigations involving larger populations are required to confirm these findings.

## Data Availability

The raw data supporting the conclusions of this article will be made available by the authors, without undue reservation.
